# An alignment-free method to find and visualise rearrangements between pairs of DNA sequences

**DOI:** 10.1038/srep10203

**Published:** 2015-05-18

**Authors:** Diogo Pratas, Raquel M. Silva, Armando J. Pinho, Paulo J.S.G. Ferreira

**Affiliations:** 1 IEETA/DETI, University of Aveiro, Portugal

## Abstract

Species evolution is indirectly registered in their genomic structure. The emergence and advances in sequencing technology provided a way to access genome information, namely to identify and study evolutionary macro-events, as well as chromosome alterations for clinical purposes. This paper describes a completely alignment-free computational method, based on a blind unsupervised approach, to detect large-scale and small-scale genomic rearrangements between pairs of DNA sequences. To illustrate the power and usefulness of the method we give complete chromosomal information maps for the pairs human-chimpanzee and human-orangutan. The tool by means of which these results were obtained has been made publicly available and is described in detail.

Structural genomic rearrangements are a major source of intra- and inter-species variation. Chromosomal inversions, translocations, fissions and fusions, are part of the naturally occurring genetic diversity of individuals, are selectable and can confer environment-dependent advantages^1^. Chromosome rearrangements are also associated with disease, namely, developmental disorders and cancer. For example, many leukaemia patients present a reciprocal translocation between chromosomes 9 and 22, also known as the Philadelphia chromosome. This produces BCR-ABL fusion proteins that are constitutively active tyrosine kinases, contributing to tumour growth and proliferation^2^. Another striking example is the human inversion polymorphism in the 17q21 region, which contains the neurodegenerative disorder-associated gene *MAPT* (microtubule associated protein Tau). The direct oriented H1 haplotype is common and relates with increased Alzheimer’s and Parkinson’s disease risk, while the inverted H2 haplotype has higher frequencies in Southwest Asia and Southern Europe populations, particularly around the Mediterranean^3^. Recurrent inversions are found in the primate lineage, where the H2 haplotype is the ancestral state, and recent work evidences that Neanderthals and Denisovans also carried the H1 allele^5^.

How genome architecture changes contribute to speciation and which macroevolutionary events occurred through time are fundamental to understand the dynamics of chromosome evolution, and hence, the origins of species. In addition, chromosome alterations are hallmarks of cancer genomes with diagnosis and prognosis value^6^, and are also used in prenatal and postnatal clinical settings. Several insights into chromosome structure and evolution have been traditionally achieved by cytogenetic procedures such as G-banding, or molecular karyotyping approaches like fluorescence in situ hybridisation (FISH) and, more recently, array-based methods^7^. However, in some groups, such as the great apes, access to samples is often difficult, e.g. due to ethical reasons. Also, these approaches can be time-consuming, expensive, or lack resolution, as opposed to computational solutions^8^.

The advent of sequencing technology enabled the analysis of genomic sequences at nucleotide resolution. Nowadays, next-generation sequencing is bringing a substantial increase of speed, quality and reliability of the results for much less costs, although there is still promising space for improvements. The availability of sequenced genomes boosted computational methods into a new era, allowing some expensive and/or lengthy *wet lab* processes to be complemented by computational approaches^9^.

Derived scientific insights from genomic sequences, including the conserved distribution of genes on the chromosomes of different species or synteny, have been mostly explored using sequence alignments^10^,^11^,^12^,^13^,^14^,^15^,^16^,^17^,^18^,^19^, while for visualisation, a wide variety of strategies have been proposed^20^,^21^,^22^,^23^,^24^. Specifically, at a macro level the most popular are Mauve^13^, Cinteny^25^, Apollo^24^, MEDEA ( http://www.broadinstitute.org/annotation/medea), MizBee^26^ and Circos^27^, which are discussed in a recent review^28^. Although, the circle-based visualisation is becoming very popular, for detecting block alignments and re-arrangements across very similar species, such as primates, an ideogram still seems to be the best approach.

We propose a computational method to detect signatures of chromosome evolution. The method is completely alignment-free and is based on the information content of the sequences being compared. The information content itself is estimated using data compression techniques. The resulting stand-alone algorithm depends only on two parameters.

We developed a tool by means of which the proposed method can be tested in practice. The tool has been made publicly available and is described in detail. It is capable of producing an SVG image that shows the correspondence of regions between two sequences. Its performance is demonstrated with the help of several examples. Those involving synthetic sequences are intended to illustrate the underlying principles. More realistic case studies, involving prokaryotic and eukaryotic genomes, are also discussed. In particular, we obtain human/chimpanzee and human/orangutan chromosome maps.

For clarity, the potential and limitations of the tool and some of its design tradeoffs are discussed separately, following the description of the method. This separates limitations that are inherent to the method from those that are by-products of the current implementation, and that as such might be removed in future implementations.

## Method

### Creating models of the data

The immediate goal of a data compression method is to describe data as compactly as possible. The usefulness of data compression as a tool to find structure in data is perhaps less well-known^29^,^3^0.

Nevertheless, this ability is a direct consequence of how data compression works. Compression methods usually rely on statistical data models that estimate the probability of the data symbols along the sequence. Better (i.e., more accurate) statistical models tend to lead to better compressors (i.e., higher compression ratios).

Ultimately, the size of the compressed data can be seen as an estimate of the Kolmogorov (algorithmic) complexity of the original data, a fundamental yet noncomputable complexity measure closely related to information theory^31^.

Genomic data compression, now more than twenty years old^32^,^33^,^34^,^35^,^36^,^37^,^38^,^39^,^40^,^41^,^42^,^43^,^44^, has been the subject of recent review articles^45^,^46^,^47^. Typically, the compression methods rely on a combination of models that explore the redundancy found in DNA sequences, usually with models developed to handle high information content (i.e., hard to compress) regions and distinct models to handle low information content (i.e,. very compressible) regions.

The method proposed in this paper identifies small-scale or large-scale rearrangements between pairs of sequences called the reference and the target. The method applies to arbitrary sequences, and therefore the reference and the target can be as large as an entire chromosome or genome. The goal of the method is to automatically detect regions in the target sequence that have information content similar to regions found in the reference. The method yields a set of segments of the target sequence and, for each of these, the corresponding segment found in the reference sequence.

Both sequences are preprocessed such that their alphabet is 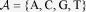. Symbols originally not belonging to 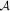 (for example, N’s) are substituted by uniformly distributed symbols from 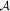, in order to keep the original length of the sequence. These random generated segments are high information content regions and, therefore, will not share information with any other sequence, hence will not interfere with the matching process.

The core of the method involves the estimation of the amount of conditional information that is required to represent a certain region of the target, using exclusively information from the reference. Basically, if *x* and *y* are, respectively, the target and reference sequences, we compute a numerical sequence 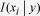, where 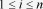 and 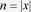 is the size of the target sequence. For a position 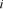 in the target sequence, 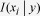 measures the number of bits required to represent the symbol located in that position, according to the aforementioned interpretation of conditional information.

To properly estimate 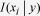, it is crucial to have a good model of the reference sequence 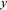. We have chosen finite-context models (FCMs) for this purpose. FCMs are probabilistic models based on the assumption that the information source is Markovian, i.e., that the probability of the next outcome depends only on some finite number of (recent) past outcomes referred to as the context.

The estimated probability distribution at position 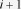, 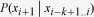, according to the order-*k* context 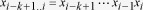 is calculated with the symbol counts previously computed on the reference sequence 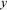, using the estimator


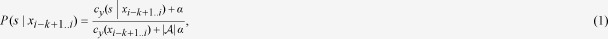


where 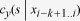 represents the number of times that symbol 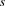 was found in sequence 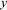 having 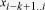 as context and where


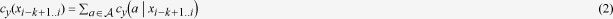


is the total number of events that occurred in *y* in association with context 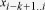. The parameter 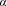 is set to 0.001, forcing the estimator to behave approximately as a maximum likelihood estimator. In practice, this makes the segmentation process easier (see below). The number of bits that is required to represent symbol 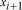 using exclusively information from the reference sequence is given by


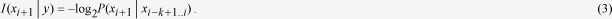


### Finding information-similar regions

**Figure 1:**
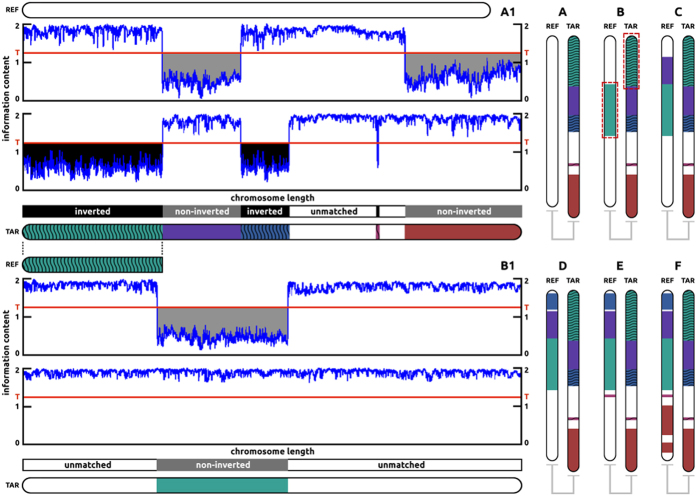
Similarity discovery, step by step. (**A**) scan the target to identify those of its regions that significantly share information content with the reference. (**B**) scan the reference to find those of its regions associated with each region identified at step **A**. Step (**C**), (**D**), (**E**), (**F**), repeat step **B** for each region identified at step **A**.

As explained before, the core idea of the method is to compute, along the target sequence 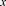, the amount of information required to represent *x* using exclusively information from the reference sequence *y*. Therefore, at a first stage, we end up with a numerical information sequence 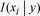 of size 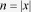. Fig 1 illustrates how the method operates, using synthetic data generated with an appropriate tool^48^. The target was created by manipulating some parts of the reference, as described in the figure. Additional examples are provided in the Supplementary Material file.

Regions where 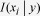 is small indicate a high level of information sharing with 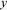. To mark them, we compare a smoothed version of the information sequence with a threshold (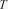). The result is the set of regions of interest of 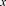, for the given reference 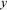, which are denoted by 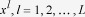.

It remains to find the regions of the reference 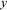 which are strongly associated with each 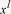. To do this we invert the roles of the reference and the target. More precisely, each 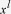 is now regarded as a reference, and 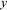 is taken as the target. We thus compute, for each 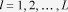, the information sequences 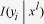, from which the regions of 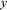 associated with each 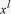 can be found.

The described procedure can find pairs of regions that are similar in the sense of information-sharing, but does not take into account possible inversions. For this purpose, the reference sequence should be reverted, complemented and loaded in the FCM model. Then steps entirely similar to those described above need to be taken. Having done this, both inversions and direct homologies can be segmented in the target sequence.

If both the inverted and direct instances of a region are found to have high information content, then the region shares no information with the rest of the data and therefore it is left unmarked. This is the case with regions that are essentially unique and with unsequenced regions (those that originally contained N’s, that have been replaced with random data).

### The tool

#### Availability

An implementation of the method (Smash) is freely available, under GPL-2 license, at http://bioinformatics.ua.pt/software/smash. Smash is a tool that computes chromosome information maps, with an ideogram output architecture. The colours for each block are automatically calculated using the HSV (Hue, Saturation, Value) colour space, where only the Hue varies. For more information about Smash, see the Supplementary Material, Section “The Smash tool”.

#### The threshold *T*

Smash has a command-line option by means of which the threshold 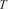 can be varied in the interval 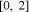 (see the Supplementary Material). The threshold can be regarded as a parameter. In general, the best 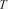 is data-dependent. The guiding principle is to choose 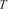 so that it selects regions of complexity sufficiently below the average. In practice, this is not difficult to achieve, but some experimentation may be required to obtain the best results.

As a rule, 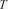 should be smaller when working with similar species than when working with more distant species. For example, for the human/chimpanzee pair we used 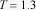 but for the chicken/turkey pair we used 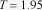. When working with entire chromosomes, the threshold can be adjusted to match the degree of divergence encountered.

### Model depth

The model depth, described by the parameter *k*, must be an integer in the range [1,28] (as described in the Subsection “Parameters, Options”, option -c. The default value (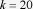) works well for sequences, say, longer than 1 Mb (1,000,000 symbols). The default also works well for smaller sequences, although in this case the actual performance may depend on how repetitive they are. We have found out that there is often little practical need to tune *k*.

The relation between the model depth *k* and the estimated probabilities (which are directly related to the counters 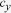), and the capabilities of Markov models in the context of DNA sequence modelling, have been treated in detail elsewhere^44^.

### Commutativity

The proposed method is fully commutative, that is, it has the potential to lead to the same results when the reference and the target are swapped. Smash can easily be made commutative as well. However, in most usage scenarios, there is a natural reference sequence. Furthermore, the assumption that one of the two sequences is the reference simplifies the algorithm and leads to time savings. For these two reasons, the current implementation of Smash is approximately commutative, but not exactly so.

To illustrate this, we performed additional experiments using both prokaryotic and eukaryotic genomes. For the prokaryotes, we have used *Shigella flexneri* (NC_017328) and *Escherichia coli* (NC_017638). As can be seen in Supplementary Fig. 2, the maps are very similar (apart from some differences in colour and reversed pattern assignment, due to the automatic colouring method used). Nevertheless, it is possible to spot small differences, mainly because we have discarded matched regions smaller than 20 kb. Supplementary Fig. 3, which illustrates the human/chimp pair, shows that at a larger scale these small differences tend to disappear.

### Working with distant genomes

Smash does work for more distant genomes than, say, the human/chimpanzee pair studied in detail next. This is shown e.g. by the chicken/turkey map of chromosome 1 included as Supplementary Fig. 1. According to TimeTree^62^, *Gallus gallus* and *Meleagris gallopavo* have an estimated divergence time of 44.6 million years (MY), while between *Homo sapiens* and *Pan troglodytes* or *Pongo abelii* the divergence times are estimated as 6.3 MY and 15.7 MY, respectively.

We emphasise, however, that Smash can be applied to pairs of sequences that are even more distant. Regardless of the exact nature of the reference and target, Smash will find the rearrangements present, even if one or both sequences are synthetic (computer generated). This can be useful to develop a better understanding of how Smash works, or for testing purposes. Examples are presented in Supplementary Figs. 4 and 5, where synthetic sequences containing different rearrangements were processed with Smash. For comparison purposes, the output of widely used tools such as Mauve^13^ and VISTA^15^ is also provided. In Supplementary Figs. 6 and 7, the methods are compared in real prokaryotic and eukaryotic sequences, respectively.

### Working with unassembled sequences or assembling errors

One of the advantages of Smash is that it works even when the reference is not assembled. Therefore, it can be used with references composed of non-assembled reads obtained directly from the NGS sequencers. In fact, although next-generation sequencing made low cost high speed sequencing possible, it also decreased the size of sequencing reads^61^. On the other hand, most of the primate assembled sequences use the human genome as a reference. This might be problematic, because of the assumption that humans and the other primates exhibit a high degree of homology, which might not always be true^53^. Hence, it might be important to measure similarity against non-aligned references.

**Figure 2:**
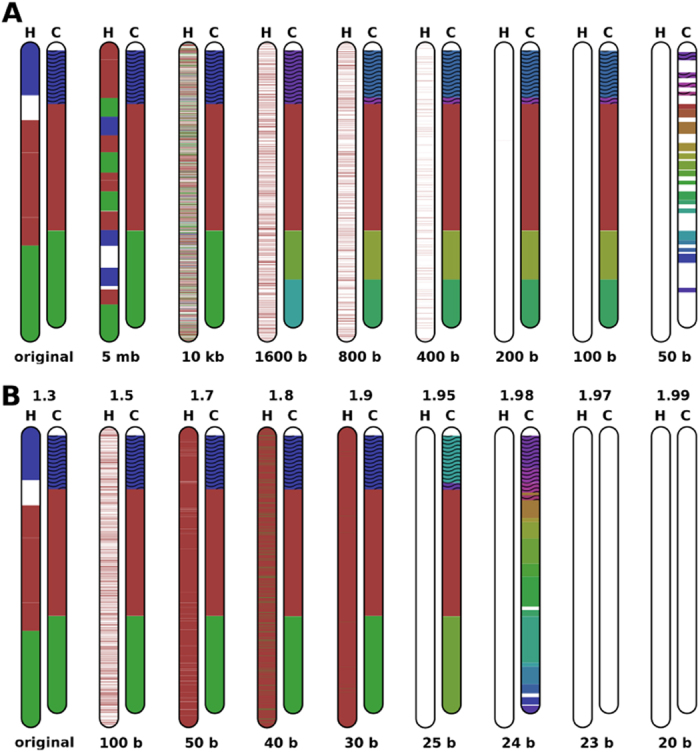
Smash computation over P. troglodytes chromosome 18, using as reference permuted blocks of different sizes from *H. sapiens* chromosome 18. Colours are only consistent for each run of the tool and, therefore, may not be consistent from one run to another run, where the sequences or the parameters are changed. (**A**) Smash was executed using 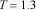 and 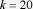. (**B**) Smash was executed using a variable threshold 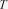 (upper value) and 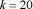.

Figure 2 depict the results of Smash over chromosome 18 of human and chimp using random permutations of blocks with different size, showing its robustness when fragmented references are used. Smash spent less than 8 minutes for each computation.

Smash is able to identify regions containing shared information even when one of the sequences is block-permuted, a capability that may be of interest to measure sequence similarity, e.g. when one of the sequences is not assembled, or when there are assembly errors. Obviously, the identification of the precise genomic rearrangements that took place will have to be deferred until final assembly takes place.

## Results and Discussion

**Figure 3:**
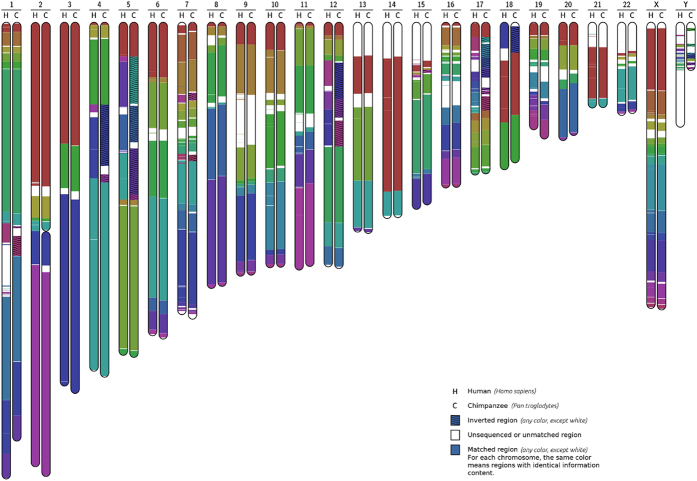
Human chimpanzee chromosomal map, obtained from chromosome pairwise comparison. Inversions can be observed in chromosomes 1, 4, 5, 7, 12, 15, 17, 18, and Y. Chromosomes 2A and 2B of chimpanzee have been fused for a more concise representation.

**Figure 4:**
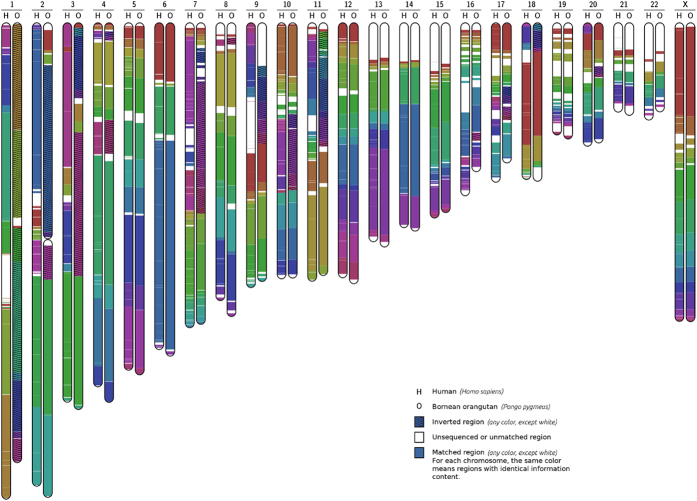
Human orangutan chromosomal map, obtained from chromosome pairwise comparison. Inversions are present in chromosomes 2, 3, 4, 7, 8, 9, 10, 11, 16, 17, 18 and 20. Chromosomes 2A and 2B of orangutan have been fused for a more concise representation.

To illustrate the potential of the proposed method, we show the complete chromosomal information maps for the pairs human-chimpanzee and human-orangutan. Additional examples can be found in the Supplementary Material. The *Homo sapiens*, *Pan troglodytes* and *Pongo abelii* reference assembled chromosomes were downloaded from the NCBI. In order to create the human-chimpanzee map, we have concatenated chromosomes 2A and 2B of the chimpanzee, ran Smash once per chromosome (totalling 23 runs), then manually corrected the associated picture regarding the hypothetical centromere between 2A and 2B, and finally grouped all the maps in one global picture (the one shown in Fig. 3). A similar process was done for the human/orangutan map, shown in Fig. 4. The results obtained confirm and extend previous work based on orthologous gene distribution, array comparative genomic hybridisation (array CGH) and FISH approaches^49^,^50^,^51^.

**Figure 5:**
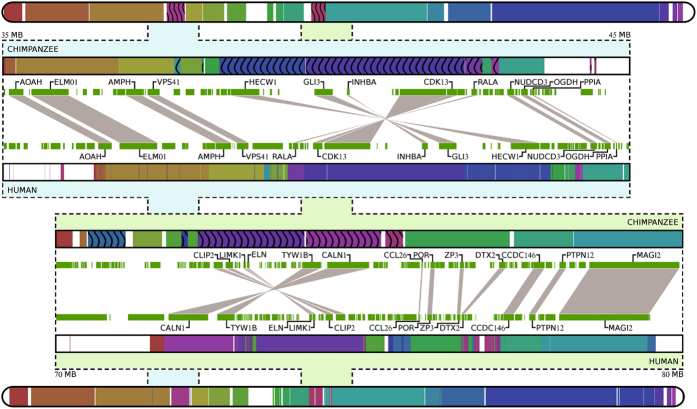
Progressive human and chimpanzee chromosome 7 information maps. For each chromosomes two regions have been extracted (35 MB to 45 MB and 70 MB to 80 MB). The progressive maps for these sub-regions show the genes involved in the paracentric inversions detected.

Figure 3 shows the complete information maps between human and chimpanzee genomes, using chromosome pairwise comparisons, which are characterised by several inversions, in chromosomes 1, 4, 5, 7, 12, 15, 17, 18, and Y. All known pericentric inversions were detected by our method with the exception of inversions in chromosomes 9 and 16 that are located in regions with limited available sequence information^52^. The structural rearrangements observed in the chimpanzee Y chromosome agree with previous reports^53^, where variable copy number and position of Y-specific genes was found among chimpanzees (*Pan troglodytes*) but not among bonobo (*P. paniscus*), gorilla (*Gorilla gorilla gorilla* and *G. beringei graueri*) or orangutan (*Pongo pygmaeus* and *P. abelii*) lineages^54^. In addition, we identify inversions in chromosome 7 (Fig. 5) that were only partially described before^50^. Despite their importance, inversions are traditionally difficult to detect and new experimental approaches have been recently developed to improve the available tools^55^. These two inversions are located in 7p14.1 and 7q11.23 around the *GLI3* and *ELN* genes, respectively, and both are associated with human disorders. Namely, the Greig cephalopolysyndactyly syndrome is caused by mutations, deletion or rearrangements in the region containing the *GLI3* transcription factor that affect the development of the limbs, head and face, and is characterised by the presence of extra fingers or toes^56^. The Williams-Beuren syndrome (WBS) is a neurodevelopmental disease with distinctive facial and behavioural features, as well as several degrees of intellectual disability, caused by deletions of genes including *ELN*^57^. Curiously, inversion polymorphisms are present in a significant proportion of parents from WBS patients^57^,^6^0, which is also observed in the 17q21.31 region^59^, suggesting that structural variants enhance some microdeletion syndromes. Given the structural differences observed in these chromosomal regions, one might speculate that they have contributed to evolutionary innovation and the emergence of lineage-specific phenotypes.

Figure 4 depicts the complete information maps between human and orangutan. It shows that orangutan chromosome 1 is in the opposite direction as compared with human. Moreover, there are large inversions in chromosomes 2, 3, 4, 7, 8, 9, 10, 11, 16, 17, 18 and 20. Although there are fewer data available, the results are consistent with previous cytogenetic approaches that identified new rearrangements on the orangutan genome, specifically, a pericentric inversion on chromosome 1, complex rearrangements on chromosome 2 and a subtelomeric deletion on chromosome 19 60. Also, recent evidence suggests that the orangutan genome maintains the ancestral chromosomal state with observable differences in most chromosomes when compared with humans, including chromosomes 1, 2, 3, 7, 10, 11 and 18 49.

The method and the implementation here described allows the detection of large-scale and small-scale genomic rearrangements, including balanced translocations and inversions that are not detected by array-CGH or chromosome alterations that are below the limits of microscopy, thus, extending the possibilities of genome-wide structure characterisation with a single tool.

In Supplementary Figs. 8 and 9 we provide an example of a translocation between chromosomes 5 and 17 of human and gorilla. As it can be seen, after concatenating the sequences, Smash was able to detect a well known translocation that is one of the bases of gorilla speciation foundations^63^.

Smash compares pairs of sequences. These pairs can be built using single chromosomes, as shown in Figs. 3 and 4, or sets of chromosomes concatenated in a single sequence, as in the example of the translocation shown in Supplementary Figs. 8 and 9. In either case, Smash looks for and reports the position of regions that are similar, from the point of view of information content. Hence, in the examples provided in Figs. 3 and 4, only the regions that are similar in each pair of chromosomes are reported. To have a full view, it would be required either to run Smash in each possible pair of chromosomes (i.e., all possible pairs formed between the set of human chromosomes and the set of chimpanzee chromosomes, or by concatenating in a single sequence the whole genome of each species). Naturally, when very large sequences are involved (for example, entire genomes concatenated), the visualization granularity is reduced and the computational resources increase. A more detailed discussion can be found in Section 2 of the Supplementary Material.

## Conclusion

Chromosome rearrangements can drive adaptation and evolution of novel traits, but they can be deleterious as well. Here, we show that compression-based models are remarkably capable of detecting signatures of genomic chromosomal evolution, namely to determine how information flows between sequences. The method is alignment-free and universal, in the sense that it can accept any input pair of genomic sequences, and depends only on two parameters.

A tool that implements the method has been made available for download. General guidelines have been given on how to select the values of its two parameters, which do not affect its performance in an overly sensitive way. Its advantages and limitations have been discussed.

The tool and the ideas that underlie its design may lead to new insights about important genomic questions, since it allows blind unsupervised detection of rearrangements and similarities between genomic sequences. An obvious example is the detection of evolutionary patterns across species, as demonstrated in the examples, but the tool has similar potential for diagnosis and genetic counselling. The detection of rearrangements in cancer genomes at high resolution levels is also considered important, in connection with risk stratification and personalised therapeutics.

## Author Contributions

D.P., A.P. and P.F. designed the algorithms. D.P. implemented and tested the software. D.P., R.S., A.P. and P.F. designed the experiments and interpreted the results. D.P., R.S., A.P. and P.F. wrote the manuscript. All authors reviewed the manuscript.

## Additional Information

**How to cite this article**: Pratas, D. *et al.* An alignment-free method to find and visualise rearrangements between pairs of DNA sequences. *Sci. Rep.* **5**, 10203; doi: 10.1038/srep10203 (2015).

## Supplementary Material

Supplementary Information
