# Supplementary material for: An alignment-free method to find and visualise rearrangements between pairs of DNA sequences
Source: Sci Rep. 2015 May 18;5:10203. doi: 10.1038/srep10203 (PMC4434998; doi:10.1038/srep10203)
Supplement: Supplementary Information [file srep10203-s1.pdf]

Supplementary Material of

"An alignment-free method to find and visualise rearrangements between pairs of DNA sequences"

D. Pratas, R. M. Silva, A. J. Pinho & P. J. S. G. Ferreira

IEETA/DETI, University of Aveiro

1 Additional examples

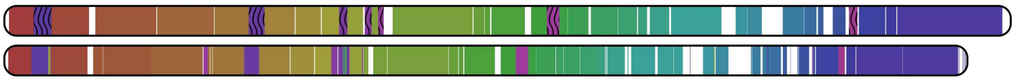

Figure 1: Smash computation of the *M. gallopavo* (top map) and *G. gallus* (bottom map) chromosome 1. Smash has been computed using a threshold $T = 1.95$, a context size of $k = 20$ and discarding blocks smaller than 1 Mb.

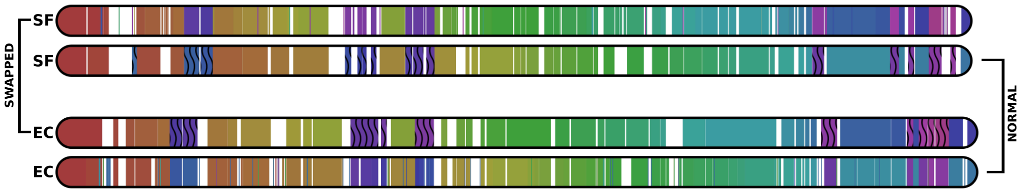

Figure 2: Smash result when the reference and the target are swapped. "SF" stands for *S. flexneri* and "EC" for the *E. coli* bacterial genomes. Smash was ran using $T = 1.8$, $k = 20$ and discarding blocks smaller than 20 kb.

Smash has the advantage of being fully capable of dealing with data coming directly from high throughput sequencing [1], a characteristic usually not found in methods based on alignments. As an example, Mauve [2], that is known to be one of the most flexible methods, was unable to create a valid map for permutations of blocks as large as 1Mb and even unable to provide any output at all (due to several crashes).

In addition to this, the running times are essentially proportional to the number of matches. Thus, when working with Smash it is irrelevant if the reference sequence is

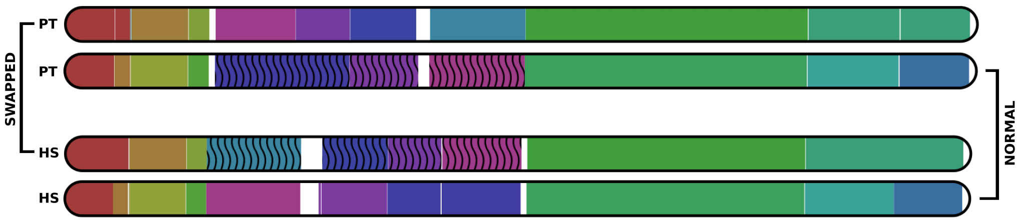

Figure 3: Smash result when the reference and the target are swapped. “HS” denotes the *H. sapiens* whereas “PT” indicates the *P. troglodytes* eukaryotic genomes. Smash was ran using $T = 1.3$, $k = 20$ and discarding blocks smaller than 1 Mb.

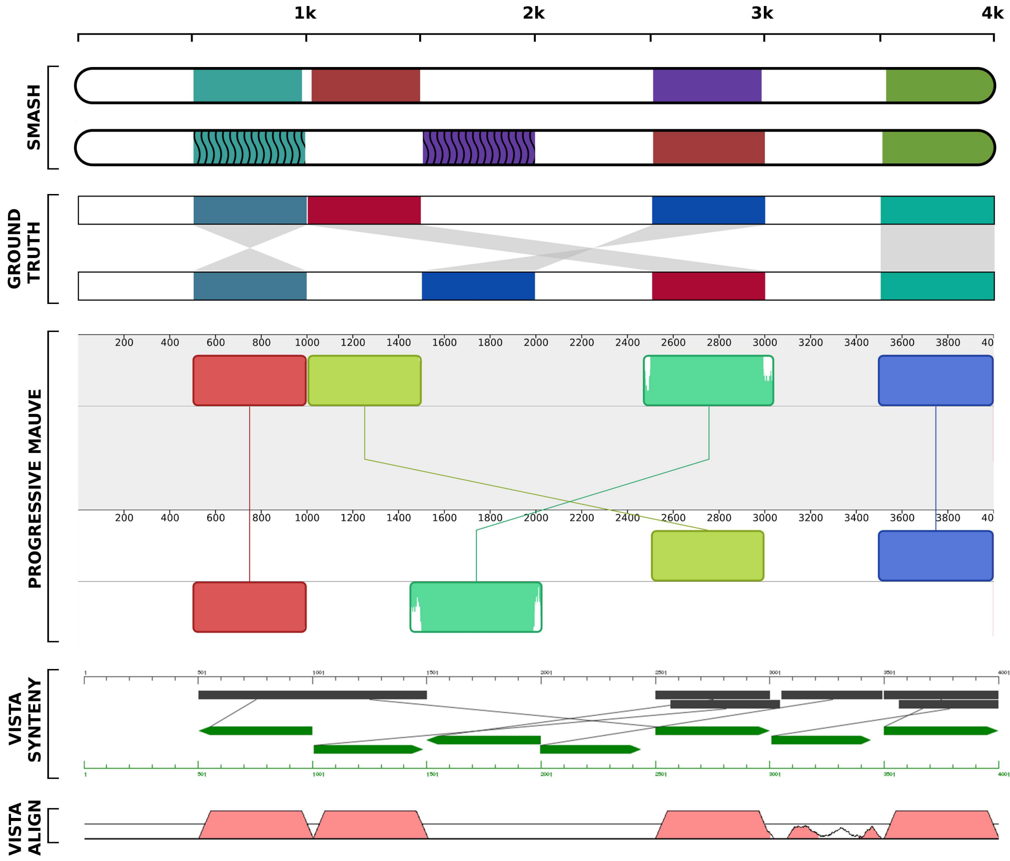

Figure 4: Comparison between Smash, Mauve and the VISTA methods on a synthetic sequence (4,000 bases) with a ground truth. Smash was ran with $T = 1.5$, $k = 10$ and discarding blocks smaller than 5 bases. VISTA was computed online, for both synteny and alignments.

shuffled (as in a NGS output). In the case of alignment methods this can be a problem (regarding running time and peak memory), even when running in computer clusters.

We have included two synthetic sequences of 4 kb each and made a comparative study

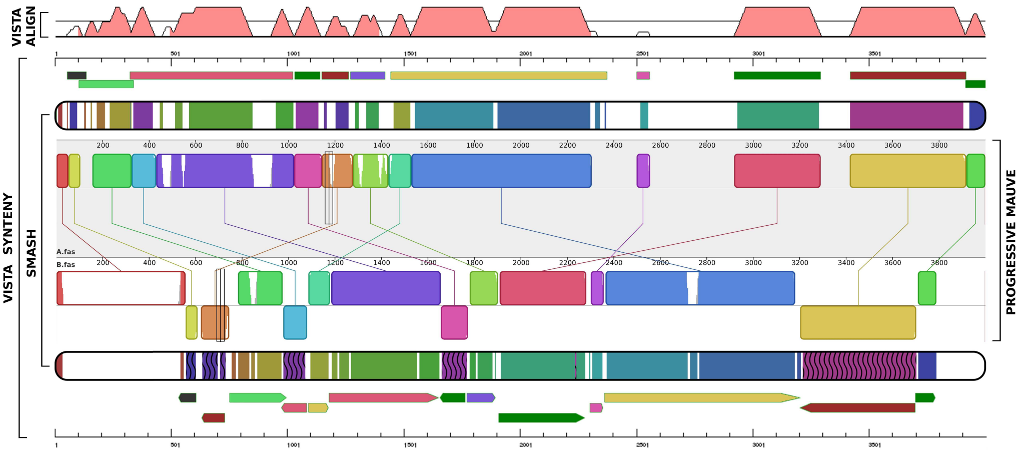

Figure 5: Comparison between Smash, Mauve and the VISTA methods on a more complex synthetic sequence (4,000 bases). Smash ran with $T = 1.5$, $k = 10$ and discarding blocks smaller than 5 bases. VISTA was computed online (syntheny) and the maps were adapted (colors instead of pointer lines) for better display. First sub-image depicts only the VISTA alignments.

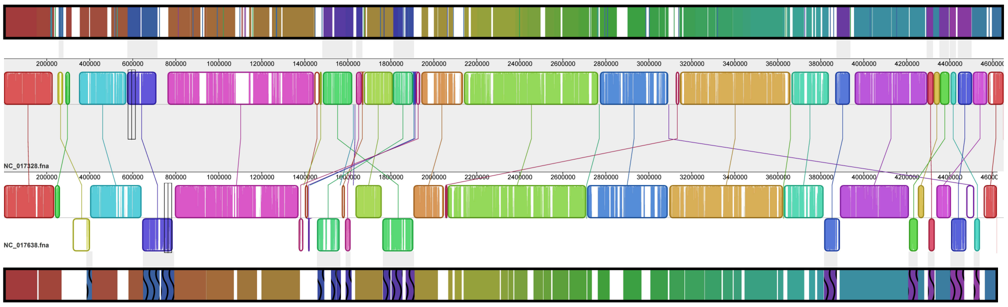

Figure 6: Comparison between Smash and Mauve methods on *S. flexneri* and *E. coli* bacterial genomes. Smash was ran using $T = 1.8$, $k = 20$ and discarding blocks smaller than 20 kb.

with respect to other techniques: Mauve [2], and VISTA [3]. The synthetic sequences of Supplementary Figs. 4 and 5 were simulated using [4] and randomly permuted using different block sizes and inversions. The first one (Supplementary Fig. 4) contains a small number of block permutations and inversions to allow an easy comparison with the provided ground truth. The second one contains overlaping permutations and, therefore, is more complex.

Supplementary Fig. 6 addresses a large-scale comparative study between *Shigella flexneri* (NC_017328) and *Escherichia coli* (NC_017638), using Smash and Mauve.

Regarding the eukaryotic example, we have used chromosome 3 of human and orangutan, depicted in Supplementary Fig. 7. Smash spent 871 seconds (for the inside Smash map) and 1291 seconds (for the outside Smash map) using a maximum memory peak of 2.4 GB,

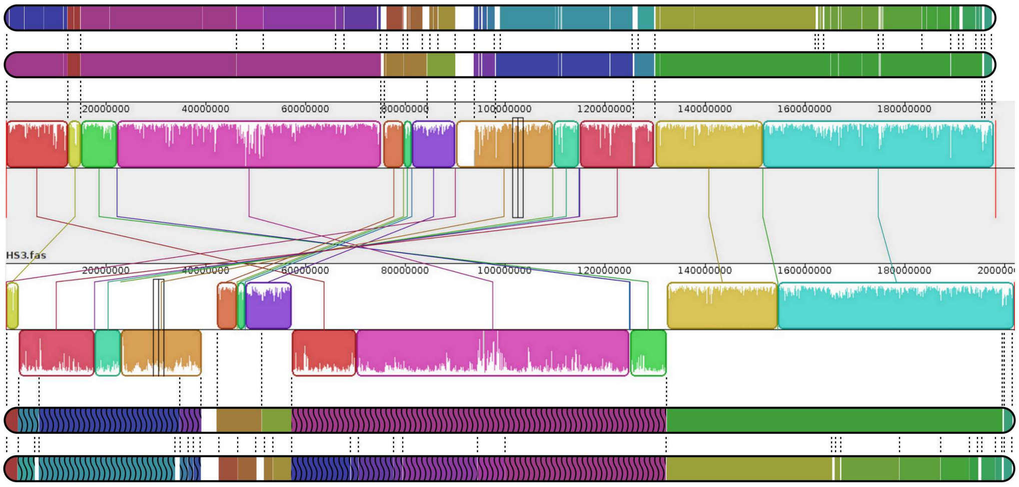

Figure 7: Comparison between Smash and Mauve methods on chromosome 3 of *H. sapiens* and *P. abelii*. Smash was ran using $T = 1.5$ (inside Smash map) and $T = 1.6$ (outside Smash map), $k = 20$ and discarding blocks smaller than 1 Mb.

while Mauve spent 2633 seconds and used 6.1 GB of memory. The Mauve version used is already the improved (latest) version.

All the experimental results have been computed on a common laptop (running Linux Ubuntu 12.04 LTS with the following hardware characteristics: 4 Intel Core i7-3520M CPU at 2.90GHz, 8 GB of RAM and a SSD of 243.6 GB). Nevertheless, the RAM memory usage never surpassed 3.1 GB, even for the larger chromosomes.

For the human/chimp maps, using $k = 20$ (the default value) and $T = 1.3$, Smash needed 212 minutes. We recall that by using properly estimated parameters the elapsed time might substantially decrease.

1.1 Translocation detection example

In Supplementary Figs. 8 and 9 we show how that Smash detects a well known translocation between human and gorilla chromosomes 5 and 17 [7]. Scripts that allow reproducing these results are included in the package containing the source code of Smash.

Specifically, in Supplementary Fig. 8 we present the detection results provided by Smash both for the case where the whole concatenated genome of the gorilla is used as reference (top) as well as when used as target (bottom). In the first case (top map), the $\approx$ 3 GB of reference required $\approx$ 22 GB of memory to run. We can clearly identify the region with homology to the human chromosome splited in two major blocks, placed in the regions corresponding to the positions of chromosomes 5 and 17 of gorilla (the precise locations can be obtained from the files produced by Smash). The bottom map shows similar results, but at the cost of considerable less resources (just 2 GB of memory and 115 minutes of computing time), because in this case the shortest sequence played the role of reference sequence.

Supplementary Fig. 9 provides more detail regarding this translocation, because in this case only chromosomes 5 and 17 of both species were concatenated and hence considered.

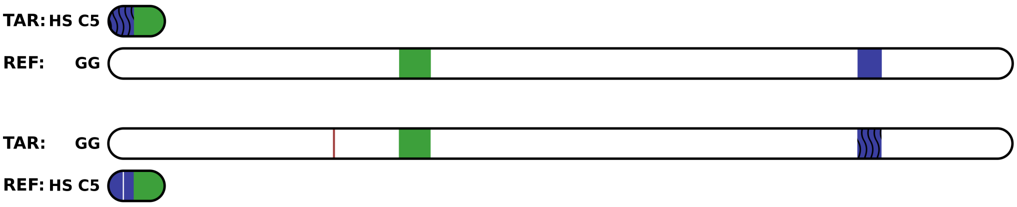

Figure 8: Detection of a translocation between the whole genome of gorilla, GG (all chromosomes concatenated), and human chromosome 5, HS C5. Smash was ran using the default parameters, twice. In the first case, GG was used as the reference and HS C5 as the target (it required ≈ 22 GB of memory and ≈ 224 minutes), while in the second case HS C5 was used as reference and GG as the target (requiring ≈ 2 GB of memory and ≈ 115 minutes to run).

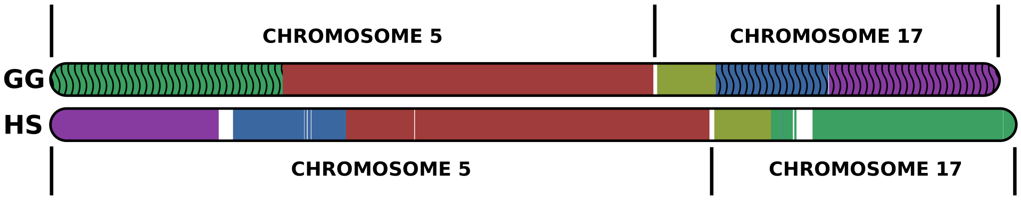

Figure 9: Detection of a translocation between gorilla and human chromosomes 5 and 17. The sequences have been concatenated (chromosome 5 and 17) in each species. In the middle of each concatenation we introduced one million of N symbols to facilitate the location of the concatenation breakpoint. Smash was ran using the default parameters.

2 Additional notes

Smash can be executed in different ways. Suppose that we are interested in comparing the sequences a_1, a_2, a_3 with b_1, b_2, b_3, b_4. Obviously, the tool can be run on each of the (a_i, b_j) pairs. Alternatively, we could form a new sequence a by concatenating all the a_i, another sequence b by concatenating all the b_i, and then run the tool (just once) on the (longer) a and b sequences. There are many other possibilities. For example, we could apply the tool to each of the pairs (a_i, b). Or, swapping a and b, one could focus instead on the pairs (b_i, a).

Regardless of how one decides to organise the analysis, the tool will detect the relations between the given sequences and will generate appropriate log files that identify with precision the type of relation, the file offsets etc.

The colors used by the tool are visual clues. They are helpful to visualise and understand the nature of the rearrangements, but for very precise results (offsets etc.) one would have to refer to the log files. The results, modulo border effects at the edges of the sequences, will be essentially the same — regardless of the approach used.

Working with concatenated sequences such as (a, b) can be helpful for exploratory analysis, when the nature of the relations is totally unknown. The results of this "large scale analysis" may expose interesting relations between parts of a and b, which can then be analysed in detail by checking specific pairs (a_i, b_j).

In our experience, after acquiring some experience with the tool, the user will have no problem in organising the analysis. The best approach depends on the main goal:

whether to check for precise relations within two relatively small sequences, between one or more small sequences and a large (possibly concatenated) one, or between two concatenated sequences.

3 The Smash tool

3.1 Installation

We provide a binary for each 64 bits operating systems (Linux, OSX, Windows). However, for other purposes, such as source code compilation, use the following installation instructions. Cmake is needed for installation (http://www.cmake.org/), although for Linux there is a possibility to skip its usage. You can download it directly from http://www.cmake.org/cmake/resources/software.html or use an appropriate packet manager. In the following instructions we show the procedure to install, compile and create the information map between human and orangutan chromosome 20:

3.1.1 Step 1

Download, install Smash and resolve conflicts.

Linux:

```
sudo apt-get install cmake
wget https://github.com/pratas/smash/archive/master.zip
unzip master.zip
cd smash-master
cmake .
make
```

Alternatively, you can install (without cmake and only for Linux) using

```
wget https://github.com/pratas/smash/archive/master.zip
unzip master.zip
cd smash-master
mv Makefile.nix Makefile
make
```

OS X:

```
# Install brew:
ruby -e "$(curl -fsSL
https://raw.github.com/Homebrew/homebrew/go/install)?"
# only if you do not have it. After type:
brew install cmake
brew install wget
brew install gcc48
wget https://github.com/pratas/smash/archive/master.zip
unzip master.zip
cd smash-master
cmake .
make
```

With some versions you might need to create a link to cc or gcc (after the brew install gcc48 command), namely

```
sudo mv /usr/bin/gcc /usr/bin/gcc-old # gcc backup
sudo mv /usr/bin/cc /usr/bin/cc-old   # cc backup
sudo ln -s /usr/bin/gcc-4.8 /usr/bin/gcc
sudo ln -s /usr/bin/gcc-4.8 /usr/bin/cc
```

In some versions, the gcc48 is installed in /usr/local/bin, therefore you might need to substitute the last two commands by the following two:

```
sudo ln -s /usr/local/bin/gcc-4.8 /usr/bin/gcc
sudo ln -s /usr/local/bin/gcc-4.8 /usr/bin/cc
```

Windows:
In windows use cygwin (https://www.cygwin.com/) and make sure that it is included in the installation: cmake, make, zcat, unzip, wget, tr, grep (and any dependencies). If you install the complete cygwin packet then all these will be installed. After, all steps will be the same as in Linux.

3.1.2 Step 2

Download the sequences [the links might change over time].

Linux and **OS X**:

```
wget ftp://ftp.ncbi.nlm.nih.gov/genomes/H_sapiens/
Assembled_chromosomes/seq/hs_ref_GRCh38_chr20.fa.gz
wget ftp://ftp.ncbi.nlm.nih.gov/genomes/Pongo_abelii/
Assembled_chromosomes/seq/pab_ref_P_pygmaeus_2.0.2_chr20.fa.gz
```

3.1.3 Step 3

Unzip, exclude headers and filter content.

Linux:

```
zcat hs_ref_GRCh38_chr20.fa.gz | grep -v ">" > HS20
zcat pab_ref_P_pygmaeus_2.0.2_chr20.fa.gz | grep -v ">" > PA20
```

OS X:

```
gzcat hs_ref_GRCh38_chr20.fa.gz | grep -v ">" > HS20
gzcat pab_ref_P_pygmaeus_2.0.2_chr20.fa.gz | grep -v ">" > PA20
```

3.1.4 Step 4

Run Smash.

Linux, **OS X** and **Windows**:

```
./smash -v -c 20 -t 1.5 HS20 PA20
```

This step outputs a SVG image using the respective map under the name: HS20PA20.svg (for custom name use option: -o ANYNAME.svg).

3.2 Parameters

The Smash program has many options in the interface because there is a wide variety of parameters that can be defined by the user. However, for the detection of the arrangements only two are critical, namely context and threshold. Additional information about these parameters can be found in the paper.

To see the possible options type

```
./smash
```

or

```
./smash -h
```

These will print the following options:

```
Usage: smash <OPTIONS>... [FILE] [FILE]

 -h                    give this help,
 -V                    display version number,
 -v                    verbose mode,
 -f                    force (be sure!),

 -c  <context>         context order (DEF: 20),
 -t  <threshold>       threshold [0.0,2.0] (DEF: 1.5),
 -m  <mSize>           minimum block size (DEF: 1000000),

 -i                    do not show inversions,
 -n                    do not show regulars,
 -r  <ratio>           image size ratio (DEF: MaxSeq/150),
 -a  <alpha>           alpha estimator (DEF: 1000),
 -s  <seed>            seed for random 'N',
 -w  <wSize>           window size,
 -wt <wType>           window type [0|1|2|3] (DEF: 0),
 -d  <dSize>           sub-sample (DEF: 10000),
 -nd                   do not delete temporary files,
 -wi <width>           sequence width (DEF: 25),

 -p  <posFile>         output positions file,
 -o  <outFile>         output svg plot file,

[refFile]              reference file,
[tarFile]              target file.
```

By default, Smash has many parameters assigned in order to avoid the estimation, enabling only to set both reference and target files. However, these defaults are only partially estimated to detect rearrangements in primates. Therefore, for other purposes you might need to adjust context and threshold parameters. Moreover, for custom image maps you might also need to set other parameters, such as width. Only [refFile] and [TarFile] are mandatory.

3.3 Options

Parameters	Meaning
-h	It will print the parameters menu (help menu)
-V	It will print the Smash version number, license type and authors.
-v	It will print progress information such as positions of the patterns, times, etc.
-f	It will force to write over files already created.
-c <context>	Size of the FCM (Markov) context order (interval [1;28]). Contexts above 14 will be handled with a hash-table, where the implementation is approximately linear in memory relatively to the size of the sequence. When the sequence is very fragmented, or the species are somehow distant, or the sequencing/assembly process has low quality this value show not be very high.
-t <threshold>	It will be used to segment the high from the low regions of information content (interval [0;2]). For distant species this value might be slightly below 2 (such as 1.9).
-m <mSize>	Minimum size of the block considered as a valid patters after each segmentation process. Values below 1 Million for primate chromosomes might emerge excessive valid patterns. However for other purposes, such as gene scale analysis, this value should be set almost to 1.
-i	it will not show inversions.
-n	it will not show regular patterns (normal).
-r <ratio>	Sets the ratio size of the image. Currently it is calculated to MaxSeqSise/150. For the same scale attribution you should set a value, such as 1000000, which is an estimated value of the medium of the primate chromosomes sizes relatively to the medium of the screen resolution. For smaller sequences, perhaps 50000 should be a good value.
-a <alpha>	Probabilities estimator. This value relates a linear interpolation between the maximum likelihood estimator and the uniform distribution. This also shows that when the total number of events is large, the estimator behaves as a maximum likelihood estimator. Default value is set to 1000.
-s <seed>	This is a parameter to the pseudo-random generation function. Different seed values ensure different generated values.
-w <wSize>	The window size among with the sub-sampling is calculated automatically, nevertheless this value might be adjusted for special needs.
-wt <wType>	Window filtering type. Types: 0, 1, 2 or 3. Type 0 stands for Hamming, 1 for Hann, 2 for Blackman, while 3 represents a rectangular window. For a common example see page 4 of [5] or through several pages in [6].
-d <dSize>	Sub-sampling value. This value among with the window size is calculated automatically. Nevertheless, for special purposes this value might be adjusted.
-nd	Smash will conserve (no delete) all temporary files.
-wi <width>	Thickness of the image for each sequence. Default value is set to 25.
-p <posFile>	The positions from all the rearrangements detected on the run (see subsection "Some considerations" for more information).
-o <outFile>	The output SVG image filename. The default uses the concatenation of reference with the target filenames (adding the "svg" extension). Beware: if the files are not in the working directory this might have problems due to several types of characters (such as '/').
[refFile]	The reference filename.
[tarFile]	The target filename.

3.4 Some considerations

The coordinates of the different blocks, as well as the direction and the respective block code, are registered in the ".pos" file, separated by tab symbols, with the following order and meaning:

- type: reference or target;
- block code (to link reference with target);
- initial position of the block;
- final position of the block;
- direction of the block (inverted or regular);

For example,

```
TARGET      1   1        39612    0-regular
REFERENCE   1   1        39612    0-regular
TARGET      2   2287593  2366817  0-regular
REFERENCE   2   2267787  2307399  1-inverted
```

indicates two similar regions. The first (code:1), of regular type, has similar information in the first 39612 bases of both sequences. The second one (code:2), inverted, shares information, respectively, from position 2287593 to 2366817 and from 2267787 to 2307399, between both sequences.

Nevertheless, to read only the target positions, there is allways the possibility of using the output of the program with the "-v" mode (verbose). In this case, it outputs several types of information including, for example, the following:

```
Running IR pattern 3 with size 13881450
Extracting pattern [20808750;34690200]
```

This example shows that there is an inverted region with size 13881450 (with starting position 20808750 and final position 34690200) that is valid, which means that its size is larger or equal than the minimum region (-m flag) that we want to consider.

3.5 License

The license is GPL v2.
For more information: http://www.gnu.org/licenses/gpl-2.0.html.

References

[1] Kircher, M. Analysis of high-throughput ancient DNA sequencing data. *Methods Mol. Biol.* **840**, 197–228 (2012).

[2] Darling, A. E., Mau, B. & Perna, N. T. progressivemauve: multiple genome alignment with gene gain, loss and rearrangement. *PLOS ONE* **5**, e11147 (2010).

[3] Frazer, K. A., Pachter, L., Poliakov, A., Rubin, E. M. & Dubchak, I. VISTA: computational tools for comparative genomics. *Nucleic Acids Res.* **32**, W273–W279 (2004).

[4] Pratas, D., Pinho, A. J. & Rodrigues, J. M. XS: a FASTQ read simulator. *BMC Res. Notes* **7**, 40 (2014).

[5] Pinho, A. J., Pratas, D. & Ferreira, P. J. S. G. Bacteria DNA sequence compression using a mixture of finite-context models. In *Proc. of the SSP* (Nice, France, 2011).

[6] Pinho, A. J., Garcia, S. P., Pratas, D. & Ferreira, P. J. S. G. DNA sequences at a glance. *PLOS ONE* **8**, e79922 (2013).

[7] Samonte, R. V. & Eichler, E. E. Segmental duplications and the evolution of the primate genome. *Nat. Rev. Genet.* **3**, 65–72 (2002).
